# Mr. Adams on the Egyptian Ophthalmia

**Published:** 1813-04

**Authors:** William Adams

**Affiliations:** Late Surgeon of the West-of-England Infirmary for curing Diseases of the Eye, instituted at Exeter. Albemarle-street


*To the Editors of the Medical and Physical Journal.*

GENTLEMEN,

THE violence and extensive dissemination of the purulent inflammation of the conjunctiva, under the appellation of Egyptian ophthalmia, has made it too generally known to require now any description of it: and, though it has certainly become less destructive since very copious bleedings have been employed, yet it has seldom, if ever, been completely subdued at its commencement by this or any other practice. Under this state of practical knowledge, in the treatment of a disease, the rapidity of whose progress we have so often occasion to witness, it became a desideratum to obtain some remedial process which should arrest the morbid actions before the eye had sustained any serious injury. The success which has attended a mode of treatment suggested by me for several persons laboring under this disease in the St. Pancras Work-house, has made me hope that this desideratum is now obtained.

The Egyptian ophthalmia has existed, in a most active and virulent state, among the children in the above establishment, for nearly two years; and had infected the attending surgeon and the nurses. During this period, the modes of treatment recommended in the publication of an eminent oculist, were tried to their fullest extent, under the superintendence of himself and his son; and, though the violence of the complaint was reduced, it was not eradicated.

On

On my being officially requested, by the acting committee of this parochial establishment, to undertake the treatment of these patients, I stated to the attending surgeons of the house, Messrs. Uppom and Lewis, of Warren-street, Fitzroy-square, that some facts had come within my knowledge, which led me to believe that this alarming disease might be stopped in its progress by the energetic use of emetics. Mr. Lewis, under whose care the ophthalmic patients principally came, undertook to superintend the experiment in the first cases of the acute form of the disease which should be brought to the infirmary.

The process was simply to give such a quantity of Antim. Tart. as would keep up constant sickness and vomiting for eight or ten hours; at the same time applying within the eye-lids some of the Ung. Hydrarg. Nitri. Oxyd. This succeeded perfectly. Vomiting was then tried without the ointment, and was equally successful.

The following extract from a document, written by Mr. Lewis, states a series of facts explanatory of the process, and its success.

"During the first fortnight of the present month (January 1813), thirteen patients with the Egyptian ophthalmia, were admitted into the infirmary of the St. Pancras Work-house. The treatment suggested by Mr. Adams was immediately put in practice, and perfectly succeeded in removing the disease in a few hours in every case, except one, that of John Kenny. This man had the ophthalmia three months since. By large bleedings and blisters, his eyes were preserved, and the acute inflammation subdued; but the disease of the inner membrane of the eye-lid still remained, and every trifling cold caused a relapse of violent inflammation. At this time he had an attack of the acute disease in one eye. In less than eight hours after the method proposed by Mr. Adams was employed, the inflammation was completely removed. A few days after, the other eye became similarly affected. For five days he perversely delayed the methods which had preserved his other eye; extensive ulceration of the transparent cornea took place, and vision in this eye was entirely destroyed.

"In all these cases, the extreme pain which attended the onset of the disease, together with the rapidly increasing inflammation, became almost immediately arrested, by Mr. Adams's mode of treatment, and the patients have been, generally, discharged from the infirmary in two or three days, with their eyes in as healthy a state as they were before the attack of the disease."

Since

Since the above was written, Mr. Lewis has employed this practice, with similar success, both in private practice and in the infirmary.

The powerful results of this practice, I think, fully entitle it to general consideration; and I hasten to lay them before the public with the view of inducing its extensive adoption, and of obtaining, through the medium of your Journal, the information of that success which I anticipate.

The only direction necessary is to exhibit the Antim.Tart. in doses adapted to the age and constitution of the patient, *as soon after the commencement of the disease as possible.* The ulcerative process frequently begins in ten or twelve days after the accession of inflammation; and it is evident that the remedy, when that stage of the disease has begun, must be ineffectual, as is strongly exemplified in the case of *John Kenny*.*

WILLIAM ADAMS,
Late Surgeon of the West-of-England Infirmary for curing Diseases of the Eye, instituted at Exeter.
*Albemarle-street, March* 16, 1813.

* The importance of this communication, containing a practical fact of such decisive utility, we trust will make a due impression on our readers; and that every opportunity will be taken to ascertain how far its efficacy extends. In the instance before us, which we hope the experience of other practitioners will fully confirm, we see a disease of the most alarming nature, rapid in its progress, and speedily destructive in its results, arrested at its onset, and all its subsequent evils prevented!—EDITORS.

