# Corrigenda

**Published:** 1813-05

**Authors:** 


### CORRIGENDA.

*Page* 304, *line* 13, *for* "ten or twelve days," *read* "ten or twelve hours."

